# To Prescribe or Not to Prescribe: A Quality Improvement Project in Improving Resident Doctors’ Confidence in Managing Acute Behavioural Disturbance

**DOI:** 10.1192/bjo.2025.10329

**Published:** 2025-06-20

**Authors:** Zuhair Ahmad, Rooble Ali, Deenan Edward

**Affiliations:** 1 Surrey and Sussex Healthcare NHS Trust, Redhill, United Kingdom; 2 Surrey and Borders Partnership NHS Foundation Trust, Redhill, United Kingdom

## Abstract

**Aims:** The management of acute behavioural disturbances necessitates an appreciation for the potential methods, risks, and monitoring requirements needed following assessment and initiation of management. A previous quality improvement project highlighted variability in clinicians initiating rapid tranquillisation agents in response to the same clinical vignette. This study aimed to improve Resident doctors’ confidence in deciding to use pharmacological or non-pharmacological methods in managing acute disturbance by 25%.

**Methods:** Initially, a fishbone diagram was created to help visualise the possible causes contributing towards lack of confidence in managing acute behavioural disturbance via word-of-mouth conversations. Subsequently, a quantitative survey was circulated amongst 25 resident doctors in a single district general hospital. The survey consisted of questions using a 5-point Likert Scale, with scores of ‘1’ representing ‘no’ and ‘5’ representing ‘extremely’. Following this, a teaching session was organised as part of the local foundation programme teaching series to help clarify common queries. A second questionnaire was then circulated, and feedback was gained to investigate changes in confidence, as well as inform future interventions.

**Results:** A total of 25 people had completed the baseline questionnaire. Confidence in utilising non-pharmacological approaches improved by 21%. Confidence in prescribing in acute disturbances improved by 32%. Overall confidence in managing a delirious patient improved by 26%.

**Conclusion:** Post-intervention, Resident doctors’ confidence in managing acute disturbances improved by 26%. Following feedback, a poster has been developed, and Resident doctors’ confidence will be re-audited.

